# Optimization of Inulin Hydrolysis by *Penicillium lanosocoeruleum* Inulinases and Efficient Conversion Into Polyhydroxyalkanoates

**DOI:** 10.3389/fbioe.2021.616908

**Published:** 2021-03-01

**Authors:** Iolanda Corrado, Nicoletta Cascelli, Georgia Ntasi, Leila Birolo, Giovanni Sannia, Cinzia Pezzella

**Affiliations:** ^1^Department of Chemical Sciences, University of Naples Federico II, Complesso Universitario di Monte Sant’Angelo, Naples, Italy; ^2^Department of Agricultural Sciences, University of Naples Federico II, Naples, Italy

**Keywords:** response surface methodology, simultaneous saccharification and fermentation, inulin hydrolysis, polyhydroxybutyrate, inulin, biorefinery

## Abstract

Inulin, a polydisperse fructan found as a common storage polysaccharide in the roots of several plants, represents a renewable non-food biomass resource for the synthesis of bio-based products. Exploitation of inulin-containing feedstocks requires the integration of different processes, including inulinase production, saccharification of inulin, and microbial fermentation for the conversion of released sugars into added-value products. In this work paper, a new microbial source of inulinase, *Penicillium lanosocoeruleum*, was identified through the screening of a fungal library. Inulinase production using inulin as C-source was optimized, reaching up to 28 U mL^–1^ at the 4th day of growth. The fungal inulinase mixture (*PlaI*) was characterized for pH and temperature stability and activity profile, and its isoenzymes composition was investigated by proteomic strategies. Statistical optimization of inulin hydrolysis was performed using a central composite rotatable design (CCRD), by analyzing the effect of four factors. In the optimized conditions (T, 45.5°C; pH, 5.1; substrate concentration, 60 g L^–1^; enzyme loading, 50 U g_substrate_^–1^), up to 96% inulin is converted in fructose within 20 h. The integration of *PlaI* in a process for polyhydroxyalkanoate (PHA) production by *Cupriavidus necator* from inulin was tested in both separated hydrolysis and fermentation (SHF) and simultaneous saccharification and fermentation (SSF). A maximum of 3.2 g L^–1^ of PHB accumulation, corresponding to 82% polymer content, was achieved in the SSF. The proved efficiency in inulin hydrolysis and its effective integration into a SSF process pave the way to a profitable exploitation of the *PlaI* enzymatic mixture in inulin-based biorefineries.

## Graphical Abstract

Scheme of the developed bioprocesses.

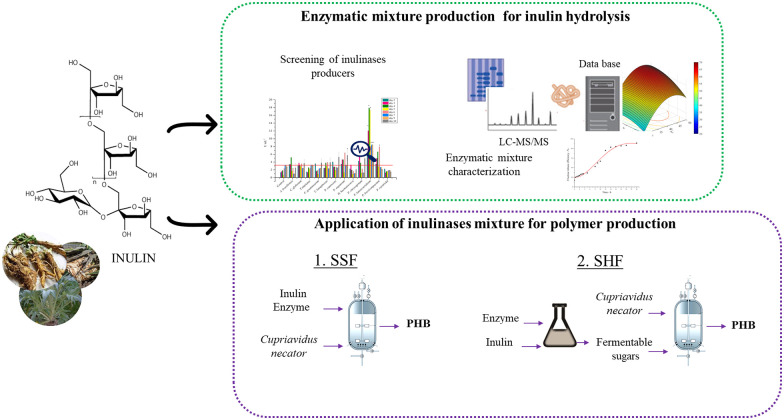

## Introduction

The biorefinery concept focuses on the sustainable conversion of renewable biomasses into a broad range of industrial products, materials, and energy. Inulin-rich biomasses represent inexpensive, renewable, and abundant feedstock to build up a biorefinery strategy (Li et al., 2013; Hughes et al., 2017; Bedzo et al., 2020). Inulin is a linear polysaccharide (ß-2,1-linked d-fructose residues terminated by a glucose residue) accumulated as a storage carbohydrate in plants such as chicory and dahlia and, more interestingly, in low-requirement crops, such as *Jerusalem artichoke* and *Cynara cardunculus* (Hughes et al., 2017). Growing well in non-fertile and harsh lands, these inulin-containing biomasses do not compete with grain crops for arable land and have received attention as renewable resource for the production of several bio-based products through microbial bioprocesses (Chi et al., 2011; Qiu et al., 2018; Singh et al., 2019, 2020a).

Among the emerging bio-based products, bioplastics derived from microbial processes, polyhydroxyalkanoates (PHA), represent promising “green” alternatives to conventional plastics. Synthesized by a wide range of bacteria from renewable sources, PHA are fully biodegradable polyesters, exhibiting a wide spectrum of properties, very close to those of fossil-derived polyolefins (Samui and Kanai, 2019; Medeiros Garcia Alcântara et al., 2020). PHA properties are dependent on their monomer chain length (ranging from the most common C4 monomer, butyrate, to C ≥ 6 monomers) with a composition strictly influenced by the supplied carbon source and the specific metabolic pathway activated in the cell. This variability translates into a wide range of material properties allowing this polymer to find applications in different sectors (Raza et al., 2018; Zhong et al., 2020). The use of low-cost substrates as starting feedstock for microbial fermentation therefore represents the keystone to promote a cost-effective and sustainable exploitation of this class of biopolymers (Kumar et al., 2019; Tsang et al., 2019; Vastano et al., 2019; Guzik et al., 2020; Surendran et al., 2020).

Hydrolysis into fermentable monosaccharides is a prerequisite for inulin utilization as carbon and energy source in the subsequent fermentation processes. Acid hydrolysis is a common method to achieve fast and cheap inulin conversion into fermentable sugars; however, it results in the formation of colored by-products as well as in the production of inhibitors of microbial growth. Enzymatic processes, conversely, besides representing an environmentally friendly alternative to acid hydrolysis, prevent the formation of pigments and inhibitors, paving the way to combined hydrolysis and fermentation steps, with an advantage in terms of overall time, costs, and productivity of the process (Qiu et al., 2018). The key enzymes involved in inulin hydrolysis are inulinases, which are glycosyl hydrolases, belonging to the GH32 family, that catalyze the hydrolysis of fructans. Based on their differential hydrolytic activity, they can be classified into exo-inulinases (E.C. 3.8.1.80), acting by removing fructose moieties from the non-reducing end of inulin, or endoinulinases (E.C. 3.2.1.7), which randomly break any β-2,1 glycosidic bond in the inulin molecule, thus releasing inulotrioses (F3), inulotetraoses (F4), and other IOSs oligosaccharides (Hughes et al., 2017). While exoinulinases have been shown to display a significant amount of activity toward sucrose, endoinulinases lack invertase activity (Chi et al., 2009; Singh et al., 2019, 2020b).

Inulinases have been reported to be present in plants, animals, and various microorganisms. The latter are the most preferred source of inulinases due to their easier manipulation, higher production levels, and variety of their properties (Singh and Chauhan, 2016, 2017). Numerous fungal, yeast, and bacterial strains have been isolated for their ability to produce inulinases (Rawat et al., 2017b; Singh et al., 2019); fungal inulinases are the most attractive for their production levels, low substrate requirement, and tolerance to low pH and high temperature (Singh and Singh, 2017). As a matter of fact, microbial inulinases have been widely exploited: exo-inulinases, in high-fructose syrup preparation (Rawat et al., 2017a; Singh et al., 2017) and endo-inulinases in the production of inulo-oligosaccharides (IOS) as functional probiotics (Singh and Singh, 2010; Chikkerur et al., 2020; Singh et al., 2020b, 2021).

Despite the high potential, the high price of commercial inulinases and the lack of efficient inulinase catalyzed processes still represent the main limitations to their effective exploitation (Qiu et al., 2019b). The cost-competitiveness of inulinase production can be achieved by using a cheap inulin-rich feedstock for their production, and several examples have been reported in this field (Singh et al., 2019). On the other hand, search for new, better-performing enzymes by exploring the potential of new inulinase producers represents a viable strategy to promote the exploitation of these enzymes in industrial processes as well as in the valorization of inulin-rich biomasses.

The production of different microbial products, including ethanol, butanol, citric and succinic acids, and single-cell proteins (Singh et al., 2020a), has been achieved while exploiting microbial inulinases in separate hydrolysis and fermentation (SHF) and simultaneous saccharification and fermentation (SSF) processes. Only few microbial strains are naturally endowed with the ability of both hydrolyzing inulin and converting fructose into value-added chemicals. Consolidated bioprocesses (CBP) based on biomass-derived inulin have been reported for the microbial production of lactic acid (Choi et al., 2012), ethanol (Khatun et al., 2017), poly-(γ-glutamic acid) (Qiu et al., 2019a), exopolysaccharide (Meng et al., 2021), and single-cell oils (Zhao et al., 2010). On the other hand, PHA production from inulin has been less explored. *Cupriavidus necator*, one of the most widely known PHA producers, is able to accumulate polyhydroxybutyrate (PHB) with high productivity from fructose but lacks the ability to utilize inulin as C-source (Bhatia et al., 2018). As a fact, only examples of SHF processes for PHA production from inulin extracts have been reported for this strain (Koutinas et al., 2013; Haas et al., 2015).

In the perspective of designing processes for saccharification of inulin substrate, the optimization of inulinase reaction conditions is essential for their utilization in the SHF process, while expanding the pool of available inulinases will help to address the specific process conditions imposed by the SSF process (Zheng et al., 2018b).

In this work, a collection of fungi was screened as inulinase producers. The inulinase mixture obtained from the best-performing fungus was characterized and used to optimize a protocol for inulin hydrolysis through an approach of statistical design of experiments. The fructose-containing hydrolyzate was used as starting feedstock for the production of PHAs from *C. necator* in an SHF process. Furthermore, the exploitation of the inulinase mixture in an SSF strategy for PHA production was also tested and the performances of the two processes were compared.

## Materials and Methods

### Microbial Strains and Culture Conditions

Fungal strains were obtained from the *Mycotheca Universitatis Taurinensis* (MUT) culture collection. *C. necator* DSM 428 production was used for PHB production.

All fungi were grown and maintained on Malt Extract Agar medium (MEA) (for 1 L): 20.0 g malt extract, 2.0 g peptone, 20.0 g agar, and 20.0 g glucose through periodic transfer at 28°C (40°C for *Thermomyces lanuginosus*) for 7–9 days. A minimal medium (MM) (Vries et al., 2017) supplemented with inulin at 10 g L^–1^ was used for the screening in liquid medium and for inulinase production. Inulin from chicory root, used in the culture media, was provided by Lineavi, Germany.

*Cupriavidus necator* DSM 428 strain was grown aerobically at 30°C in both rich (Tryptic Soy Broth, TSB) and minimal media (MM_Cn_) according to Budde et al. (2011a, b).

### Screening of the Fungal Library

For screening in liquid medium, all fungi were grown on an MEA plate for 7–9 days. A mycelium plug (1 cm diameter) was transferred on the MM + Inulin 1% w/v agar plate for an additional 7–10 days before the inoculum in liquid medium. Shake flasks, smooth conventional or baffled (250 mL), containing 50 mL of MM were inoculated with four mycelium plugs (0.5 cm diameter) and incubated at 28°C (40°C for *Thermomyces lanuginosus*) in an orbital shaker at 200 rpm.

### Inulinase Production From *Penicillium lanosocoeruleum*

*Penicillium lanosocoeruleum* cultures were made on an agar MM medium with 1% glucose and then transferred to a liquid medium. 1% w/v of inulin, glucose, or fructose was tested as different carbon sources for the preinoculum phase. After 3 days of growth (28°C, 200 rpm), the preinoculum was milled, tenfold diluted into 1-L baffled flasks containing the MM medium plus 10 g L^–1^ inulin, and grown for 10 days in the same conditions. Different medium/flask volume ratios were tested in the inoculum phase (1:2; 1:3, 1:5). Samples of fungal cultures were daily withdrawn and assayed for inulinase activity in the culture broth.

Different methods were tested to concentrate secreted inulinases in the crude extract: (i) precipitation by the addition of 80% (NH_4_)_2_SO_4_ at 4°C; (ii) precipitation in cold acetone at fourfold volume with respect to the sample; and (iii) ultrafiltration in Amicon^®^ Stirred Cell Millipore with a 10-kDa cutoff cellulose membrane.

Total protein content was determined according to the method of Bradford (1976) using bovine serum albumin (BSA) as standard. The concentrated *P. lanosocoeruleum* enzymatic mixture recovered after the ultrafiltration step was herein defined *PlaI*.

### Inulinase Activity Assay

Enzymatic activity on inulin and sucrose substrates was measured using the 3,5-dinitrosalicylic acid (DNS) reagent method in conformity with (Miller, 1959). A 0.2 mL of sample was added to 1.8 mL of 0.2 mol L^–1^ sodium acetate buffer, pH 5.0, containing 0.5% w/v of high purity grade inulin (provided by Sigma) or 1% of sucrose. The mixture was incubated at 37°C for 15 min. Total reducing sugars were measured by adding 3 mL DNS reagent and boiling in a water bath for 5 min. Samples were allowed to cool, and their absorbance was read at 540 nm. A calibration curve was obtained using fructose as standard. One enzymatic unit (inulinase or invertase activity) was defined as the amount of the enzyme which produces 1 μmol of reducing sugars per minute. All the assays were carried out in duplicate.

The effect of pH on inulinase activity was measured at 37°C in a pH range of 3–10.5 using 0.1 mol L^–1^ of citric acid–sodium citrate (pH 3–4), sodium acetate (pH 4.5–5.5), phosphate (pH 6–8), and sodium carbonate (pH 9.2–10.5) buffers. The effect of temperature was determined in 0.2 mol L^–1^ M sodium acetate buffer at pH 5, incubating the mixture for 15 min in the temperature range 30–80°C. Thermal stability of the *PlaI* mixture was determined by measuring the residual inulinase activity after incubation at 40, 60, and 80°C.

### Experimental Design

Inulin hydrolysis was optimized using response surface methodology (RSM) with central composite design (CCD). Four factors were selected to evaluate the response pattern and to determine the optimal combination of temperature, pH, substrate concentration, and enzyme loading. The coded values for each parameter were as follows [−1 0 1]: temperature in°C [28, 40, 50], pH [5, 6, 7], substrate concentration in g L^–1^ [40, 50, 60], and enzyme loading in enzyme units for gram of inulin [20, 40, 60]. The experimental design was developed using JMP^®^ 14.1.0 (SAS Institute Inc., 1989–2019, Cary, NC)^1^ and resulted in 26 conditions; all conditions were tested in triplicate.

### Enzymatic Hydrolysis of Inulin

Enzymatic hydrolysis was performed in 10-mL glass vials with 5 mL working volume. Different amounts of *PlaI* were added to Na-acetate (0.1 mol L^–1^, pH 5) or Na-phosphate buffer (0.1 mol L^–1^, pH 6, and 7) supplemented with inulin (high-grade purity) at the desired concentration. The vials were hermetically covered and incubated for 24 h while shaking at 250 rpm.

Kinetic of inulin hydrolysis was assessed in the optimal condition defined by DOE. Samples were withdrawn at different times and incubated at 100°C to inactivate the enzymatic mixture. For each reaction, a corresponding control was carried out in the absence of enzyme, to consider possible inulin spontaneous hydrolysis. The presence of free sugars into inulin powder without any incubation was taken at time 0 h. Conversion efficiency was calculated on the basis of maximum fructose released per gram of inulin. The complete inulin hydrolysis was carried out by incubating the *Aspergillus niger* endo-exo-inulinase enzyme mixture (SIGMA CAS: 9025-67-6) for 4 h at 50°C (5 U g_substrate_^–1^). Afterward, the mixture was kept in 100°C boiling water for 1 h to assure that the complete hydrolysis and fructose released was assayed. Concentrations of fructose and glucose were determined by D-fructose and D-glucose assay kits (K-FRUGL Megazyme).

### Gel Electrophoresis and Activity Staining on Polyacrylamide Gel

SDS-PAGE was performed according to Laemmli (1970). Native electrophoresis was carried out on 7% gel according to a method proposed by Chen et al. (1996). After that, PAGE gel was subjected to activity staining (Pessoni and Braga, 2007).

### *In situ* Digestion

The two gel bands that demonstrated inulinase activity were cut, destained, and *in situ* digested. Briefly, the gel pieces were washed with three cycles of 0.1 mol L^–1^ NH_4_HCO_3_ of pH 8.0 and acetonitrile, followed by reduction (10 mmol L^–1^ DTT in 100 mmol L^–1^ NH_4_HCO_3_, 45 min, and 37°C) and alkylation (55 mmol L^–1^ IAM in 100 mmol L^–1^ NH_4_HCO_3_, 30 min, and RT). The gel pieces were washed with three further cycles of 100 mmol L^–1^ NH_4_HCO_3_ of pH 8.0 and acetonitrile. Finally, the gel plugs were rehydrated in 40 mL sequencing grade modified trypsin (10 ng mL^–1^ trypsin; 10 mmol L^–1^ NH_4_HCO_3_) and incubated overnight at 37°C (Lettera et al., 2010). Peptide mixtures were eluted, vacuum-dried, and resuspended in 0.1% v/v formic acid for LC-MS/MS analysis.

### LC-MS/MS

LC-MS/MS analyses were carried out on a 6520 Accurate-Mass Q-TOF LC/MS System (Agilent Technologies, Palo Alto, CA, United States) equipped with a 1200 HPLC system and a chip cube (Agilent Technologies) and on an LTQ Orbitrap-XL (Thermo Scientific, Bremen, Germany) as reported in Linn et al. (2018), and raw data were analyzed as reported in Vinciguerra et al. (2015). Each LC-MS/MS analysis was preceded and followed by blank runs to avoid carryover contamination. MS/MS spectra were transformed in Mascot Generic files (.mgf) format, and the FASTA file of all the proteins from the gene expression profiling of *P. lanosocoeruleum* were used as database for protein identification.^2^(10698 sequences; 5093396 residues). A licensed version of MASCOT software^3^ version 2.4.0 was used. Standard parameters in the searches were as follows: trypsin as the enzyme; 3 as the allowed number of missed cleavages; 10 ppm MS tolerance and 0.6 Da MS/MS tolerance; and peptide charge from 2+ to 4+. In all the database searches, carbamidomethylation of cysteine was inserted as fixed chemical modification, but possible oxidation of methionine and the transformation of N-terminal glutamate or glutamine to pyroglutamate were considered as variable modifications. Only proteins presenting two or more peptides were considered as positively identified. Protein scores were derived from ion scores as a non-probabilistic basis for ranking protein hits.^4^The ion score is −10^∗^Log(P), where P is the probability that the observed peptide match is a random event. The individual ion score threshold provided by MASCOT software to evaluate the quality of matches in MS/MS data was used for the confidence threshold in protein identification. Basic Local Alignment Search Tool (BLAST) was used to calculate the sequence similarity among the amino acid sequences of the *P. lanosocoeruleum*-identified proteins with fungal proteins in the NCBI database.

### PHA Production and Extraction

*Cupriavidus necator* was grown on a rich medium (TSB) for 24 h and precultured in MM_Cn_ for an additional 48 h before the inoculum in fermentation media. PHA production was carried out in 250-mL Erlenmeyer flasks. For the SSF process, 20 g L^–1^ inulin was supplemented to the minimal medium together with both 40 and 80 *PlaI* inulinase U g_substrate_^–1^. For the SHF process, inulin hydrolyzed in the best conditions defined by DOE was used as a carbon source. The pH was adjusted to 6.8 using 1 mol L^–1^ NaOH, filtered, and added to MM_Cn_ (final concentration of fructose 20 g L^–1^). The flasks were inoculated at 0.1 OD mL^–1^ and incubated at 30°C on shaker running at 200 rpm for 5 days. Single flasks were taken every 24 h, and the cells were recovered by centrifugation at 5,500 *g* for 15 min and lyophilized for determination of the cell dry weight (cdw). Analysis of fructose concentration was carried out on culture supernatants, while the PHA polymer was extracted from the lyophilized cell according to Sayyed et al. (2009).

### ^1^H-NMR Spectroscopy

Polyhydroxyalkanoates extracts were analyzed by ^1^H-NMR spectroscopy: samples (0.5–1.5 mg) were resuspended in deuterated chloroform (500 μL). ^1^H-NMR spectra were recorded on Bruker DRX-400 (^1^H-NMR: 400 MHz) in CDCl3 (internal standard, for ^1^H: CHCl_3_ at d 7.26 ppm) (Mostafa et al., 2020).

### Statistical Analysis

The results were statistically analyzed using the JMP 14.1.0 (SAS Institute Inc., 1989–2019, Cary, NC)^1^. Arithmetic means and mean square errors (SD) were calculated in all cases. Significant differences in average values of inulinase activity measured in the liquid screening were tested using the Tukey-Kramer HSD test (significance level: *P* < 0.05). ANOVA test has been applied to the experimental results of CCRD and to model validation experiments. The interaction and quadratic effect of parameters were determined based on an alpha 0.05 using the *F* test. The fitted models were evaluated by normal probability plots, R^2^, and adjusted R^2^.

## Results and Discussion

### Screening of a Library of Fungal Strains for Inulinase Production

#### Library Screening

A library of twelve fungi was assembled by choosing among strains with reported evidence in literature of inulinase production and/or for which the presence of genes belonging to the GH32 inulinase family was deduced from querying the CAZY database^5^ (Table 1). All the strains were purchased from MUT collection, choosing, when available, those isolated from the rhizosphere or soil environment.

**TABLE 1:** Library of fungal strains constructed in this work.

**Fungal strain**	**Note**
*Penicillium brevicompactum* MUT 793	Inulinase activity detected (Abd et al., 2014) Annotated enzyme models: **4**
*Thermomyces lanuginosus* MUT 2896	Inulinase activity detected (Nguyen et al., 2013) Annotated enzyme models: nd
*Penicillium chrysogenum* MUT 618	Inulinase activity detected (Gujar et al., 2018) Annotated enzyme models: **6**
*Penicillium canescens* MUT 1158	Annotated enzyme models: **13**
*Penicillium lanosocoeruleum* MUT 3921	Annotated enzyme models: **9**
*Penicillium expansum* MUT 1164	Inulinase activity detected (Fernandes et al., 2012) Annotated enzyme models: **4**
*Penicillium raistrickii* MUT 1525	Annotated enzyme models: **5**
*Nectria haematococca* MUT 5670	Growth on inulin substrate (Battaglia et al., 2011) Annotated enzyme models: **6**
*Fusarium graminearum* MUT 209	Inulinase activity detected (Gonçalves et al., 2016) Annotated enzyme models: **5**
*Chaetomium globosum* MUT 337	Growth on inulin substrate (Battaglia et al., 2011) Annotated enzyme models: **6**
*Talaromyces stipitatus* MUT 237	Annotated enzyme models: **7** (Vries et al., 2017)
*Aspergillus brasiliensis* MUT 4853	Annotated enzyme models: **7** (Vries et al., 2017)

*Information reported in the table is from scientific literature available on strains belonging to the same genus and species and/or whose genome sequence was deposited on the JGI genome portal (https://genome.jgi.doe.gov/portal/). The number of annotated enzyme models derives from CAZY database; nd, not detected.*

**FIGURE 1:**
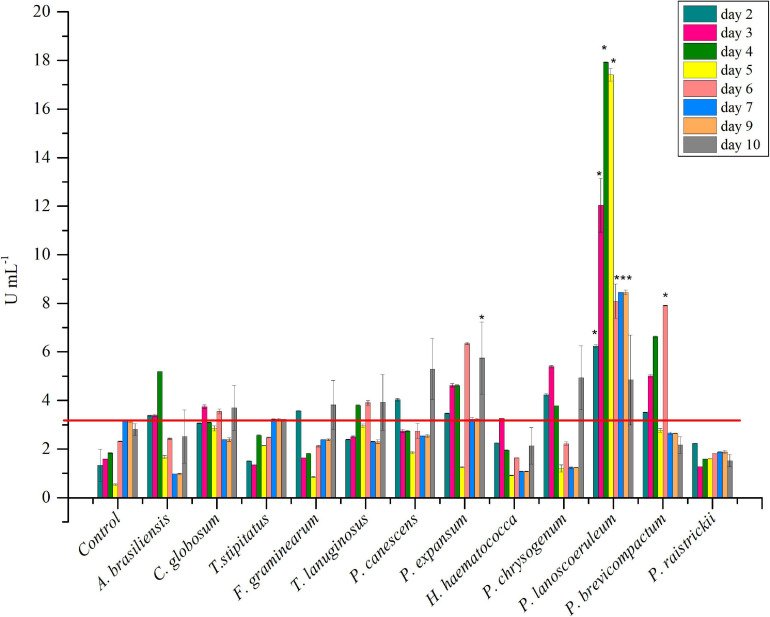
Time-course of inulinase activity as monitored in fungal culture supernatants. Each data bar represents the mean ± SD of two independent experiments. Significant values (*) (*P* < 0.05) were estimated respect to the uninoculated “Control” experiment, representative of spontaneous inulin hydrolysis (Tukey–Kramer HSD test, see Supplementary Material 1). The red line marks the maximum value of spontaneous inulin hydrolysis achieved in the same growth condition.

The strain collection was screened for inulinase production in liquid medium. *P. brevicompactum* and *P. lanosocoeruleum* exhibited the best performances. As a fact, the highest level of inulinase activity was detected in the extracellular media of the two abovementioned strains: about 18 U mL^–1^ at the 4th day and 9 U mL^–1^ at the 6th day, with an I/S ratio equal to 1 and 2, respectively, with these values being indicative of the prevalence of inulinase activity over the invertase one (Singh and Singh, 2010; Figure 1). For all the other tested strains, inulinase production does not go beyond ∼5 U mL^–1^. Based on these results, *P. lanosocoeruleum* was selected for further exploitation.

#### Inulinase Production From *P. lanosocoeruleum*

Inulinase production was carried out in liquid cultures (250 mL volume, smooth flasks) using inulin as C-source. When different medium/shake flask volume ratios were tested (1:5; 1:3; 1:2), comparable results were obtained at 1:5 and 1:3 ratios (∼15 U mL^–1^), while a significant decrease in inulinase production levels was observed at the 1:2 ratio (∼5 U mL^–1^). When the flask geometry was changed from smooth to baffled flaks, no relevant differences in terms of inulinase production levels were observed, whatever was the medium/flask volume ratio used. However, the use of baffled flasks assured more reproducible results, possibly because of a reduced formation of fungal pellets. Interestingly, when the culture was scaled up to 1 L, the use of baffled flasks yielded an almost three-fold higher inulinase production with respect to not-baffled ones (∼15 U mL^–1^ vs. 5 U mL^–1^), confirming the levels obtained on the small scale.

The type of C-source used for preinoculum growth was found to strongly affect enzyme production in the following inoculum. When the preinoculum was carried out using glucose as C-source, a notable increase in inulinase production was achieved in the following culture step with respect to inulin and fructose.

Inulinase production has been reported to be induced by the presence of inulin itself (Singh and Singh, 2017) and to be sensible to catabolite repression by free sugars (Mahmoud et al., 2011; Singh and Chauhan, 2017; Garuba and Onilude, 2020). Consistently, no inulinase production was observed when the fungus was grown in the presence of fructose or glucose as unique C-sources in the inoculum phase. Moreover, the high fructose concentration in the preinoculum (released by inulin hydrolysis, or directly available in the medium) was found to inhibit further inulinase production in the inoculum phase, while the presence of glucose (the minority monomer in inulin polymer) did not interfere with the following inulinase production (data not shown).

In the optimized condition, *P. lanosocoeruleum* inulinase activity production reached a maximum of 28 U mL^–1^ at the 4th day of growth. Inulinase production seems to be growth-associated: as a fact, a decline in enzyme activity was observed after the 4th day, possibly ascribable to the secretion of proteolytic enzymes. A similar profile was already reported for inulinase production in shake-flask fermentations of many fungal species, i.e., *Penicillium* sp. (Rawat et al., 2015b), *Aspergillus fumigatus* (Chikkerur et al., 2018), *Aspergillus niger* (Mahmoud et al., 2011), and *Aspergillus tritici* (Singh et al., 2020b).

Inulinase activity levels achieved in this work are among the highest ever obtained from submerged fermentation of *Penicillium* strains. A novel strain of *Penicillium subrubescens* (FBCC 1632^T^) isolated from soil has been found to produce up to 7.7 U/mL^−1^ when tested on pure inulin (Mansouri et al., 2013), while a production level of 1 U/mL^−1^ has been reported for *Penicillium* sp. NFCC 2768, and this strain is much more effective as inulinase producer (up to 3.9 U/mL^−1^) when grown on inulin-rich vegetable infusions with respect to pure inulin (Rawat et al., 2015b). Similarly, four *Penicillium* strains, selected from a fungal library of inulinase producers on inulin-rich plant extract, displayed a production level ranging from 0.5 to 2.7 U/mL^−1^ (Rawat et al., 2015a). A higher production level (up to 20 U mL^–1^) has been achieved by Abdal-Aziz and coauthors (Abd Allah AbdAl-Aziz et al., 2012) with *Penicillium citrinum* grown on pure inulin, by increasing the incubation temperature to 35°C. A maximum of 46 U mL^–1^ of inulinase production has been reported for *Penicillium* sp. XL10 in an inulin-containing medium after optimization of the supplied nitrogen source (Zheng et al., 2018b), while about 38 U/mL^−1^ was obtained with *Penicillium oxalicum* BGPUP-4 in a growth media containing both inulin and lactose (Singh and Chauhan, 2017).

### Characterization of the Inulinase Enzymatic Mixture

Several methods to concentrate proteins from the growth medium were tested to recover an extracellular enzymatic mixture endowed with high inulinase activity, i.e., acetone precipitation, ammonium sulfate precipitation, and ultrafiltration. The latter method provided the highest recovery of enzymatic activity (∼90%), as well as an almost doubling of the specific activity of the extract (from 453 to 905 U mg^–1^). Conversely, acetone and ammonium sulfate precipitation resulted in a dramatic drop of the recovered activity (∼10% yield of recovered activity), probably due to the high glycosylation level typical of secreted fungal proteins. The notable specific activity of the *P. lanosocoeruleum* crude extract (*PlaI*) denotes a high inulinase production ability of the strain. As a fact, the specific activity so far reported for enzymatic extracts from *Penicillium* strains ranges from 80 to 740 U mg^–1^ and has been achieved after at least two purification steps (Pandey et al., 2016).

**FIGURE 2:**
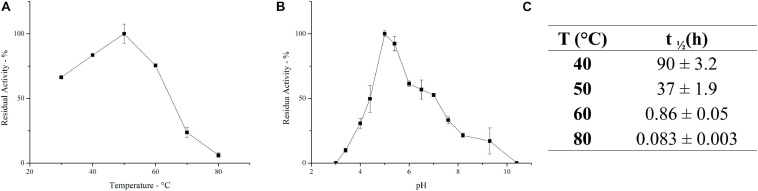
Effect of temperature **(A)** and pH **(B)** on the activity of the enzymatic mixture. **(C)** Half-life of the inulinase enzymatic mixture at different temperatures. Each data point represents the mean ± SD of two independent samples.

The ultrafiltrated broth enriched in inulinase activity, herein defined as *PlaI*, was further characterized. The effect of temperature on the activity of the inulinase mixture was determined in the range 30–80°C. *PlaI* displays a maximum at 50°C and retains >70% of its activity in the range 30–60°C (Figure 2A). The pH activity profile displays a maximum at pH 5, along with a retention of more than 50% of the enzymatic activity in the pH range 4.5–7 (Figure 2B). The heterogeneity of the enzymatic mixture may explain the deviation from a bell-shaped behavior between pH 6 and 7 (Figure 2B). The biochemical properties exhibited by *PlaI* are in agreement with the characteristics reported for most of the purified fungal inulinases, i.e., a pH optimum in a range 4–7 and a temperature optimum in the range 30–60°C (Rawat et al., 2017b).

*PlaI* thermostability was evaluated at selected temperatures, and t_1__/__2_ was calculated at each value (Figure 2C). The mixture shows a very good stability at 40°C, retaining its full activity up to 3 days of incubation. A reduction in thermal stability was observed at 50°C, although the enzymatic mixture still retains 50% of its activity after 37 h. The high activity of *PlaI* at 50°C, combined with the observed stability at this temperature, represents an extremely advantageous feature for inulin processing on an industrial scale, allowing the solubilization of inulin at high concentrations (Flores-Gallegos et al., 2015; Qiu et al., 2018). However, a drastic drop in enzyme stability was recorded at higher temperatures: at 60°C, the half-life of the enzymatic mixture is lower than 1 h, while at 80°C it sharply reduces to a few minutes.

#### Protein Characterization of the Inulinase Mixture

**FIGURE 3:**
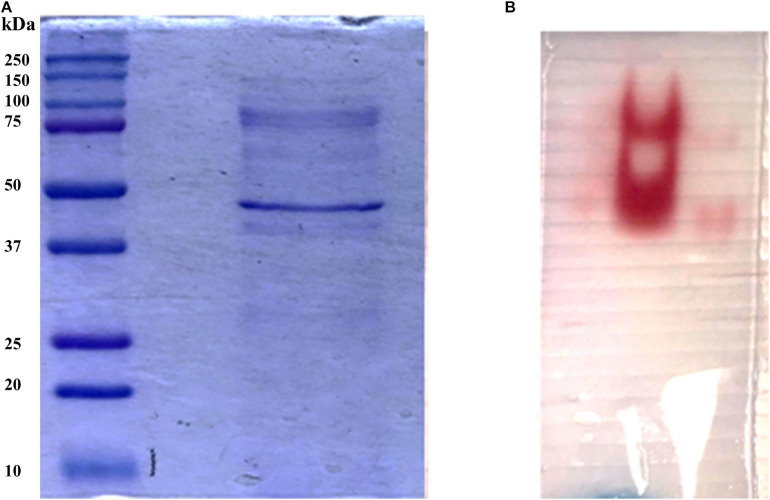
**(A)** SDS-PAGE of ultrafiltrated broth; **(B)** Zymographic analysis of a native PAGE revealed by inulinase activity.

In order to identify the proteins endowed with inulinase activity, the *PlaI* enzymatic mixture was analyzed by zymography, by staining for inulinase activity (Figure 3). The two active protein bands were detected, excised, and analyzed by an *in situ* proteomic approach, and the proteins were identified by searching raw LC-MSMS data against the set of putative proteins encoded in the annotated *P. lanosocoeruleum* genome (see text footnote 2).

**FIGURE 4:**
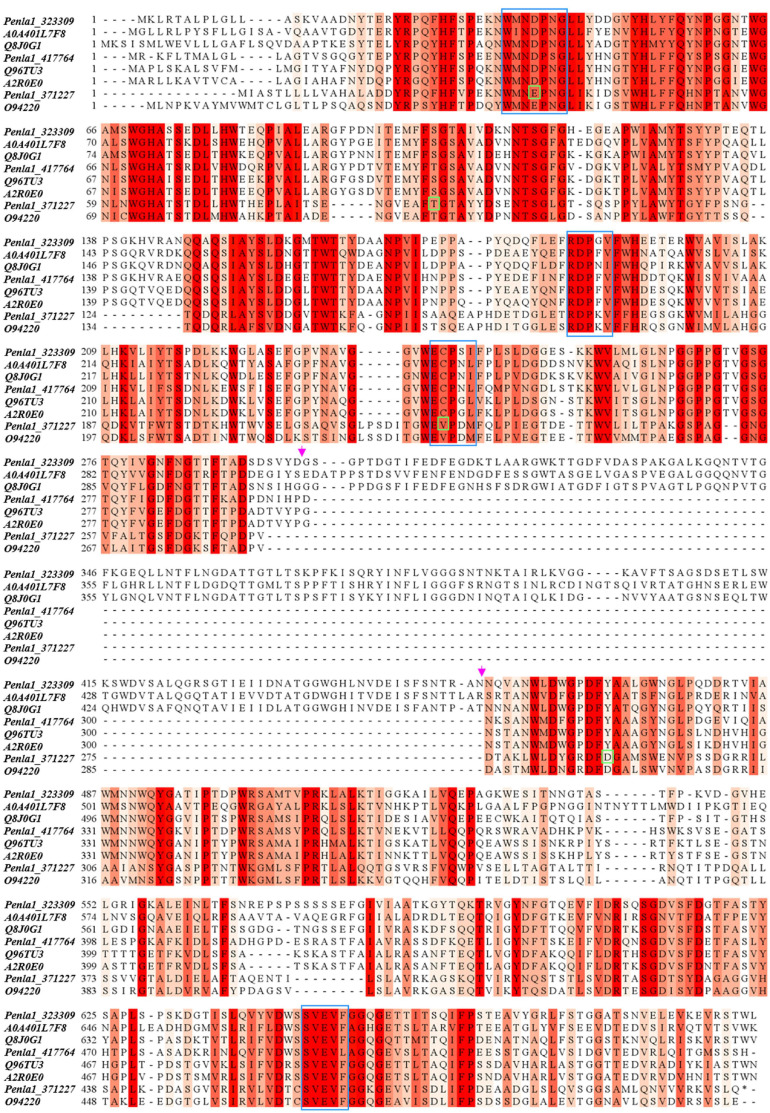
Sequence alignment of identified *P. lanosocoeruleum* inulinases with representative members of fungal exo- and endo-inulinases. The following sequences were used in the alignment: *A0A401L7:* exoinulinase from *Aspergillus awamori*; *Q8J0G1:* exoinulinase InuE from *Penicillium* sp. TN-88; *Q96TU3:* exoinulinase InuE from *Aspergillus awamori*; *A2R0E0:* exoinulinase InuE from *Aspergillus niger*; *O94220:* endoinulinase Inu2 from *Aspergillus ficuum*. The alignment was achieved using the CLUSTAL W program. Sequence conservation is shown in different shades of red. Conserved motifs of exoinulinases are highlighted by blue boxes; distinctive endoinulinase residues in green boxes. The pink arrows delimit the internal “inulin-affinity” region.

Table 2 reports the proteins identified in the two active bands (see Supplementary Material 2 for details of identified peptides). Several putative glycosyl hydrolase (GH) proteins of different families were identified in both bands. In the upper band in the gel, 10 proteins were identified; 9 proteins in the lower gel band. Three proteins (Protein ID: 323309; 417764; 371227) were found to be annotated as members of the GH32 family, with two of them present in both gel bands. When submitted to BlastP analysis, Penla1_323309 displays 74.8 and 72.7% identity with *Aspergillus fumigatus* InuD exoinulinase (Q4WDS4) and *Aspergillus lentulus* exoinulinase InuE (A0A0S7DXQ8), respectively, and is closely related to several other fungal exoinulinases. Penla1_417764 shows the highest identity (90.9%) with a putative GH32 hydrolase from *Penicillium rubens* as well as 63.4 and 62.5% identity with the exoinulinases InuE from *A. niger* (A2R0E0) and *A. awamori* (Q96TU3), respectively. From the multiple alignment with representative members of fungal exoinulinases, both Penla1_323309 and Penla1_417764 exhibit all the conserved motifs and residues characterizing this class of enzymes (Figure 4). Interestingly, the two putative *P. lanosoceruleum* exoinulinases differ for the length of an internal sequence reported to function as an additional non-catalytic inulin-affinity region in *Penicillium* sp. TN-88 InuD, responsible for a higher affinity for the substrate (Moriyama and Ohta, 2007).

**TABLE 2:** Protein identification in the active gel lanes by LC-MSMS.

**Band gel lane**	**Protein ID**	**Score**	**Number of identified peptides**	**Protein sequence coverage (%)**	**Genome annotation**
Lower gel band lane	**323309**	295	10	19	**GH32 family/GH116 family**
	**371227**	204	8	16	**GH32 family**
	376719	166	5	9	Multicopper oxidase
	327740	165	6	16	GH43 family
	383312	149	7	14	Glucooligosaccharide oxidase
	387674	122	6	10	GH64 family
	383083	120	5	16	GH17 family
	**417764**	111	4	7	**GH32 family**
	381496	62	5	11	Putative oxidoreductase
Upper gel band lane	384244	423	20	54	GMC oxidoreductase family
	315441	274	12	42	GH2 family
	400960	253	11	32	Uncharacterized protein
	**371227**	185	10	34	**GH32 family**
	385992	181	10	32	Amidase
	**323309**	177	12	33	**GH32 family/GH116 family**
	401322	99	7	18	GH20 family
	373946	147	11	28	GH3 family
	383654	145	11	31	Uncharacterized protein
	401322	99	7	18	GH20 family

*Protein bands were *in situ* digested and analyzed by LC-MSMS, and raw data was searched on the protein database as described in details in the “Materials and Methods” section. Search details and extensive description of the identified peptides are reported in Supplementary Material 2. The proteins belonging to the Gh32 family are highlighted in bold. Description of the glycosyl hydrolase (GH) families according to CAZypedia (http://www.cazypedia.org/): **GH2**: includes the following activities, β-galactosidases, β-glucuronidases, β-mannosidases, exo-β-glucosaminidases, and a mannosylglycoprotein endo-β-mannosidase; **GH3**: groups together exo-acting β-D-glucosidases, α-L-arabinofuranosidases, β-D-xylopyranosidases, N-acetyl-β-D-glucosaminidases, and N-acetyl-β-D-glucosaminide phosphorylases; **GH17**: includes two major groups of enzymes with related but distinct substrate specificities, namely, 1,3-β-D-glucan endohydrolases and 1,3;1,4-β-D-glucan endohydrolases; **GH20**: includes exo-acting β-N-acetylglucosaminidases, β-N-acetylgalactosamindase β-6-SO_3_-N-acetylglucosaminidases, and exo-acting lacto-N-biosidases; **GH32:** includes: enzymes able to hydrolyze fructose containing polysaccharides, i.e., inulinases, invertases, levanases, and other; **GH43**: includes the following major activities, α-L-arabinofuranosidases, endo-α-L-arabinanases (or endo-processive arabinanases) and β-D-xylosidases; **GH64**: laminaripentaose-producing β (1,3)-glucanases; **GH116**: includes enzymes with diverse specificities, including β-xylosidase, β-glucosidase, and N-acetylglucosaminidase; **GMC oxidoreductase:** glucose dehydrogenase/choline dehydrogenase/mandelonitrile lyase.*

Penla1_371227, instead, resulted to be related to fungal endoinulinases, displaying 74.7 and 69.6% identities with *Penicillium subrubescens* endoinulinase Inu2 and *A. niger* InuA, respectively. Consistently, the Penla1_371227 sequence reveals the presence of all the conserved motifs and the unique aminoacidic residues described for fungal endoinulinases (Chikkerur et al., 2018; Singh et al., 2020a; Figure 4).

### Inulin Hydrolysis by *PlaI* Mixture Using Response Surface Methodology

An experimental design approach was applied to investigate the effect of different parameters on the *PlaI*-catalyzed hydrolysis of inulin. In all the tested experimental conditions, inulin was efficiently converted into monomeric sugars (Table 3). ANOVA was used to determine the influence of independent variables on the dependent response. The *F* value, which substantiates the significance of the model, is 73.42, which is very high if compared to the critical value, thus indicating its significance (Supplementary Material 3). On the basis of a regression analysis, a second-order polynomial equation in terms of the coded value was generated:

**TABLE 3:** Enzymatic inulin conversion under the experimental conditions (pH, T, substrate concentration, and enzyme loading) explored in the CCRD.

	**pH**	**°C**	**Substrate concentration (g L^–1^)**	**U g_substrate_^–1^**	**Fructose (g L^–1^)**	**St. dev.**	**Conversion efficiency %**
1	5	37.5	45	27.5	39.66	0.79	73.4
2	5	37.5	45	42.5	44.06	0.64	81.6
3	5	37.5	55	27.5	45.05	1.01	68.3
4	5	37.5	55	42.5	53.23	1.07	80.7
5	5	52.5	45	27.5	36.03	0.98	66.7
6	5	52.5	45	42.5	41.24	0.50	76.4
7	5	52.5	55	27.5	47.26	0.51	71.6
8	5	52.5	55	42.5	52.81	0.24	80.0
9	7	37.5	45	27.5	35.61	0.25	65.9
10	7	37.5	45	42.5	41.95	0.62	77.7
11	7	37.5	55	27.5	37.87	0.21	57.4
12	7	37.5	55	42.5	47.70	0.23	72.3
13	7	52.5	45	27.5	24.72	0.61	45.8
14	7	52.5	45	42.5	32.77	0.08	60.7
15	7	52.5	55	27.5	35.44	1.08	53.7
16	7	52.5	55	42.5	40.17	0.77	60.9
17	4	45	50	35	45.05	0.36	75.1
18	8	45	50	35	26.36	1.07	43.9
19	6	30	50	35	35.01	1.38	58.3
20	6	60	50	35	22.99	0.01	38.3
21	6	45	40	35	42.14	1.26	87.8
22	6	45	60	35	60.00	1.08	83.3
23	6	45	50	20	43.26	0.21	72.1
24	6	45	50	50	53.01	0.59	88.3
25	6	45	50	35	47.07	1.02	78.4
26	6	45	50	35	46.03	0.42	76.7

*Fructose concentration has been determined after 24 h incubation. Conversion efficiency has been calculated as reported in the section “Enzymatic Hydrolysis of Inulin”.*

Fructose=-152.7+45.32⁢A*+6.911⁢B*-3.79⁢C*

-0.256D*-0.211A*B*-0.140A*C*+0.047*

AD*+0.031B*C*-0.006B*D*+0.007C**

D-2.885A*-20.081B*+20.038C*+20.004*D2

where A is the pH; B, the temperature (°C); C, the substrate concentration (g L^–1^); and D, the enzyme loading (U g^–1^).

The significance of each parameter’s coefficient was assessed by Prob > *F* value: values less than 0.05 indicate the significance of the model terms, and values greater than 0.1 depicts insignificance model terms. In the selected model, all the tested factors (A, B, C, D) have an effect on the hydrolysis process. The interaction effects between pH and temperature (A^∗^B) and substrate concentration and temperature (B^∗^C) were also significant (Supplementary Material 3).

The quadratic terms of pH, temperature, and substrate concentration show significant contribution to the model. *P*-value < 0.0001 suggests that pH and temperature are the experimental variables with the greatest influence on inulin hydrolysis, and therefore a small variation in their value will strongly affect the product formation rate. Moreover, the negative sign of the coded coefficients A and B suggests that the curve is concave.

The *P*-value 0.008 for the quadratic substrate concentration term indicates an effect of this variable on the hydrolysis process, although less significant than that of the pH and T. The positive sign of the relative coefficient indicates that the curve is convex; thus, the uppermost is the substrate concentration, the higher is the amount of released fructose. On the contrary, the probability value of the coefficient of the quadratic effect of enzyme loading is very high (0.337), indicating that the quadratic model does not fit with the observed data. As a fact, the contribution of this parameter is better described by a linear model. The *Lack of Fit F*-value of a model is useful to describe the co-relation between response variable and independent factors. The fitness of our experimental model is proved by a non-significant *Lack of Fit F*-value (0.543). The goodness of the models is also confirmed by the determination (*R*^2^ = 0.98) and adjusted determination coefficients (Adj.R^2^ = 0.97). Moreover, a difference < 0.2 between adjusted R^2^ and predicted R^2^ (0.95) values further substantiated the robustness of the model.

On the basis of this model, the optimal conditions to maximize inulin conversion (69.4 g L^–1^ of fructose) are as follows: T, 45.4°C; pH, 5.1; substrate concentration, 60 g L^–1^; enzyme loading, 50 U g_substrate_^–1^.

**FIGURE 5:**
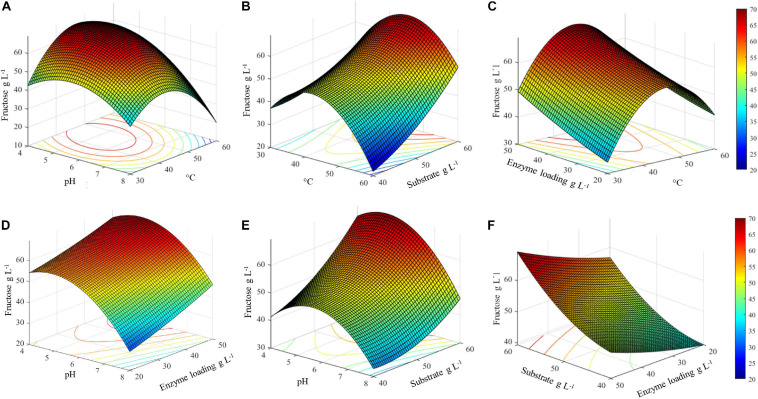
Surface plots of combined effect of process variables on inulin conversion. **(A)** Temperature and substrate concentration; **(B)** pH and substrate concentration; **(C)** pH and temperature; **(D)** pH and enzyme loading; **(E)** temperature and enzyme loading; **(F)** substrate concentration and enzyme loading.

The three-dimensional surface plots display the interaction between two independent variables on the dependent variables (fructose), while keeping the other two independent variables at their respective optimal values (45.4°C, pH 5.1, inulin 60 g L^–1^, and 50 U g_substrate_^–1^) (Figure 5). The 3D graph plots of the combined effect of temperature with three variables, i.e., pH, enzyme loading, and substrate concentration, suggest that there is an optimal temperature range between about 35 and 55°C in which the highest fructose concentration can be achieved (Figures 5A,C,E). Specifically, in this temperature range, the highest fructose release can be reached if pH is lower than 6. Consequently, a decrease in the response yield is observed out of this temperature range at pH > 6 (Figure 5A).

It is worth noting that, in the same optimal temperature range, the substrate concentration has a remarkable effect on the release of fructose, with the highest value was obtained at an increasing inulin amount (Figure 5B). Conversely, the combined effect of enzyme loading and temperature is not significant: at the optimal temperature, 45.4°C, more than 60 g L^–1^ of fructose can be obtained even when lowering the enzyme amount from 50 to 30 U g_substrate_^–1^. Within the optimal temperature range, about 50–60 g L^–1^ of fructose is released whatever is the amount of enzyme used (20–50 U g_substrate_^–1^) (Figure 5C).

The combined effect of pH and enzyme loading indicates that at the optimal pH (5.1) more than 60 g L^–1^ of fructose is obtained using from 30 to 50 U g_substrate_^–1^. However, when moving far from the optimum, a comparable response is assured by increasing the enzyme loading (Figure 5D). Similarly to what was observed for temperature, a pH optimal range (4–6.5) can also be identified, within which an increasing amount of fructose can be obtained by increasing the substrate concentration (Figure 5E). Taking together all the observed effects, it can be assumed that within the optimal range of pH and T, fructose release can be adjusted by acting on enzyme and substrate concentrations (Figure 5F). Interestingly, in the investigated range of substrate concentration, from 40 to more than 60 g L^–1^ of released fructose can be achieved by using an intermediate enzyme loading (30 U g_substrate_^–1^).

In order to validate the RSM model, experiments were carried out in the proximity of the estimated optimal conditions (Table 4). The predicted results were compared to the experimentally obtained values, and the *T*-test at 95% confidence showed no significant differences between predicted and experimental values.

**TABLE 4:** Model validation: predicted and experimental enzymatic inulin conversion around the estimated optimal conditions.

**pH**	**Temperature°C**	**Substrate concentration g L^–1^**	**Enzyme loading U g_substrate_^–1^**	**Fructose g L^–1^**	
				**Predicted**	**Experimental**
5.1	45.5	60	50	69.4	69.8 ± 1.5
5.6	38.2	60	50	65.1	64.2 ± 0.8
4.3	46.6	60	50	67.1	66.4 ± 0.6

The ANOVA (*F*-test) applied to the experimental data resulted in an *F*-value of 21.64 and *R*^2^ = 0.88. Thus, the proposed RSM model can be a useful tool to predict maximum inulin conversion.

**FIGURE 6:**
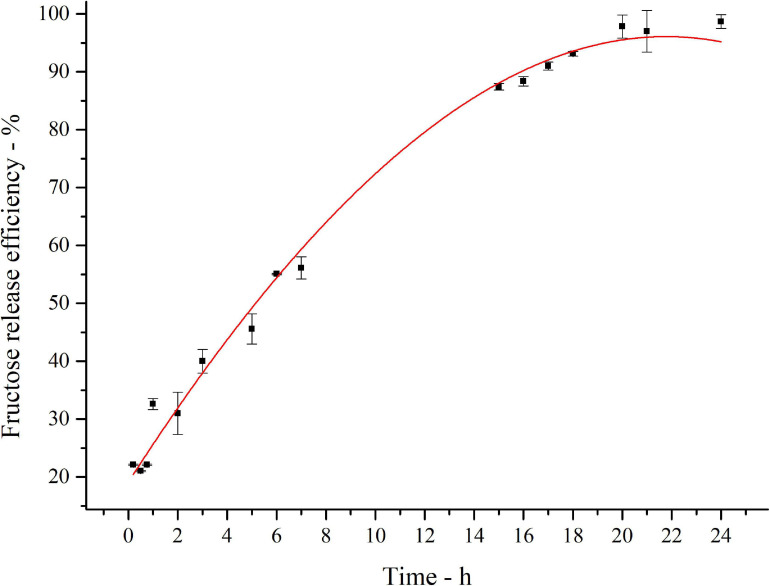
Fitted curve plot of kinetics of inulin conversion in the optimized hydrolysis condition. Regression analysis was performed with OriginLab (OriginLab Corporation, Northampton, MA, United States). The determination coefficient for polynomial fit is *R*^2^ = 0.99.

The kinetics of inulin conversion in the optimized hydrolysis condition is reported in Figure 6. The release of fructose increases progressively to reach about 60% in the first 8 h. The 90% is achieved in 16 h, settling in a plateau level of 97% of conversion after 20 h. Experimental data were fitted with a third-order polynomial model. The goodness of the predictive models is confirmed by the determination coefficient (*R*^2^ = 0.99).

Although the available literature data on inulin hydrolysis are not easily comparable because of the several variables affecting the process (source and amount of inulin, reaction conditions, determination of inulin conversion) (Sirisansaneeyakul et al., 2007; Mutanda et al., 2009; Saber and El-Nagg, 2009; Wang et al., 2013; Zheng et al., 2018a), the results obtained with *PlaI* mixture are worth of notice, since an almost complete hydrolysis at high substrate concentration (60 g L^–1^) was achieved in the optimized conditions. In a similar RSM-based approach, up to 95% fructose yield has been obtained by hydrolysis of 60 g L^–1^ Jerusalem artichoke-derived inulin, with a commercial *A. niger* inulinase (10 U/g^−1^, 48°C, pH 4.8) (Sarchami and Rehmann, 2014). The definition of optimal T and pH for inulin hydrolysis by commercial Fructozyme (Novozyme) with an RSM approach has led to a fructose yield of 82.5% after 12 h at 35°C and pH 5.2, with 30 U/g^−1^ of Jerusalem artichoke powder (Wang et al., 2013). Statistical optimization of inulin hydrolysis has been applied to immobilized inulinase from *A. tubingensis*, resulting, in the best conditions (60°C, 10 U/g^−1^, 12 h), in a hydrolysis yield higher than 70 and 85% from chicory and asparagus inulin, respectively, both supplied at 17.5% (Trivedi et al., 2015). High conversion efficiency (up to 88%) in a short time (5 h) using *P. citrinum* inulinases has been also reported, by using a lower amount of inulin (10 g L^–1^) and a very high enzyme loading (2,500 U g_substrate_^–1^) (El-Hersh et al., 2011). Similarly, a variable degree of hydrolysis, in the range 50–70%, has been shown by using a high amount of *A. tamarii* AR-IN9 inulinase (1,000–3,000 U/g) at 45°C, pH 5.2, after 2 h, depending on the agro-waste used as inulin source (Saber and El-Nagg, 2009).

### Exploitation of *PlaI* Mixture in a Process for PHA Production From Inulin

The applicability of the *PlaI* mixture was tested in a combined process of inulin hydrolysis and PHA production by *C. necator* DSM428. Previous reports have shown that high PHB accumulation in *C. necator* has been achieved starting from an initial fructose concentration of 20 g L^–1^ (Budde et al., 2011a; Koutinas et al., 2013). For the design of an SSF process, *PlaI* hydrolytic performances were preliminarily tested in conditions reproducing the *C. necator* culture media (MM_Cn_, pH 6.8, 30°C) containing 20 g L^–1^ inulin, corresponding to a maximum yield of 24 g L^–1^ releasable fructose.

When different *PlaI* amounts (10, 20, 40, and 80 U g_substrate_^–1^) were tested, the amount of released fructose after 24 h of incubation increases linearly (2.4, 3.8, 7.4, and 10.3 g L^–1^, respectively). However, all the obtained values were largely below the theoretical ones expected from a complete substrate hydrolysis. Inulinase activity measured in culture resulted to be reduced up to 42% of the initial one, indicating that both the pH and salt concentration of the growth medium affected the *PlaI* enzymatic activity.

**FIGURE 7:**
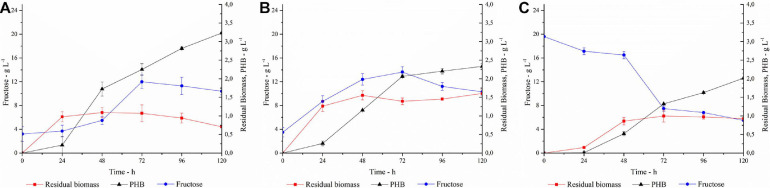
PHA production, fructose concentration and residual biomass profiles in **(A)** SSF with 40 U g_substrate_^–1^; **(B)** SSF with 80 U g_substrate_^–1^; **(C)** SHF. Each data point represents the mean ± SD of three independent experiments, carried out in duplicate.

In order to ensure an adequate amount of C-source for the microbial growth, the SSF process was carried out adding 40 U g_substrate_^–1^ (trial A) or 80 U g_substrate_^–1^ (trial B) of *PlaI* to the culture medium containing 20 g L^–1^ inulin, and the process triggered with the simultaneous inoculation of *C. necator* (Figures 7A,B). About 3 g L^–1^ of fructose was measured at the zero time, the inoculum time, corresponding to the amount of free sugars present in the inulin substrate used in these trials.

In trial A, with a *PlaI* concentration of 40 U g_substrate_^–1^, a slow increase in fructose concentration coupled to cellular growth was observed in the first 24 h, after which the cells entered the stationary phase. PHA accumulation started at 24 h and constantly increased during the stationary phase, reaching up to 3.2 g L^–1^ at 120 h, corresponding to 82% of polymer content. The fructose profile was monitored during the growth and reflects the simultaneity of the two processes, i.e., fructose release due to inulin hydrolysis and its consumption due to microbial growth. The rate of fructose release was higher than its consumption up to 72 h, determining an increase in fructose concentration. Conversely, after 72 h, fructose concentration slightly decreased, probably for a slowdown in the inulin hydrolysis, due to enzyme inactivation and/or inulin consumption. It is worth noting that the *PlaI* mixture ensures a high level of fructose during the whole process, supporting PHA accumulation, despite the unfavorable starting conditions of the growth media for its activity. Most likely, acidification of the culture media and salt consumption occurring during the microbial growth could help in restoring the conditions for optimal *PlaI* activity. When the same process was carried out in the presence of 80 U g_substrate_^–1^ of *PlaI* (Trial B), no increase in polymer accumulation was observed. As a fact, a maximum of 2.3 g L^–1^ PHA was achieved after 120 h, corresponding to a 60% polymer content. Thus, comparing the two SSF processes, a higher biomass accumulation was obtained in Trial B. The kinetic of polymer accumulation also followed a different trend in the presence of a higher *PlaI* loading, displaying a rapid increase up to 72 h, reaching an almost stationary level at 120 h. Hence, a slower and more gradual release of fructose in Trial A seems to be more favorable for PHA accumulation. In both processes, the released glucose was not utilized as C-source, in agreement with data already reported for the same *C. necator* strain (Azubuike et al., 2019). In both trials, a residual fructose settled to about 10 g L^–1^ at the end of the process, probably due to the exhaustion of other medium components, such as N-source or oxygen, as already reported by Koutinas in 2013 (Koutinas et al., 2013). Further trials carried out using a higher inulin concentration in the growth medium (30 g L^–1^) resulted in an overall inhibition of cellular growth, with about 0.8 g L^–1^ cdw at 120 h (data not shown). On the other hand, negligible PHA production (0.032 g L^–1^) and biomass levels (0.4 g L^–1^) were obtained at 120 h, in a control experiment performed in the absence of *PlaI*.

The SSF processes were compared to the SHF one (Trial C) where the best conditions for inulin hydrolysis, as determined by DOE, were applied and the resulting hydrolyzate was used as C-source for the PHA production process (Figure 7C). The pH of the hydrolyzate was adjusted to 6.8, and the fermentation was carried out with a C-source content of 2 g L^–1^ and 20 g L^–1^ for glucose and fructose, respectively. In this condition, the growth is characterized by a long lag phase, with fructose concentration decreasing slowly in the first 48 h. Polymer accumulation occurred in the stationary growth phase, reaching up to 2 g L^–1^ after 120 h, corresponding to about 62% polymer content. As observed in Trial B, the availability of high fructose level in the first growth phase promotes a lower PHA accumulation with respect to the conditions that ensure a more gradual release of fructose (Trial A).

Only few examples have been reported so far on the use of inulin-rich biomasses as renewable feedstocks for PHA production (Koutinas et al., 2013; Haas et al., 2015). Koutinas et al. used ground Jerusalem artichoke tubers as substrate in solid-state fermentation of *Aspergillus awamori*, producing a crude enzymatic mixture that was later employed in the hydrolysis of the inulin extracted from the tubers. The crude hydrolyzate was tested as fermentation medium for PHB production from *C. necator*, achieving up to 52% of intracellular PHB content and a concentration of 4 g L^–1^ of polymer (Koutinas et al., 2013). Similarly, Haas et al. described a process for the production of PHB from chicory root hydrolyzate using a commercially available inulinase mix of endo/exo-inulinases, comparing the performances of three different *C. necator* strains. Up to 78% PHB accumulation has been reported for the best-performing strain (Haas et al., 2015).

To our knowledge, this is the first example of SSF finalized to PHA production from inulin. Although it is well reported that the SSF strategy usually leads to superior productivity of the target product with respect to SHF, since it circumvents the inhibitory effect of high sugar concentration on cell growth (Ge et al., 2010; Li et al., 2014); it is worth noting that this process is effective only if fermentation conditions are also optimal for enzyme activity (Wang et al., 2013). The encouraging results obtained in shaken flasks represents a proof of concept of the exploitability of the *PlaI* mixture in the PHA production process by *C. necator*. It is expected that the implementation of the proposed SSF process in bioreactors, with fine controls of oxygen levels, will further improve its performance.

In all the conditions tested (Trials A, B, C), the polymer was extracted and analyzed by H-NMR. The spectra display the presence of three groups of characteristic signals of the homopolymer polyhydroxybutyrate (PHB) (Mostafa et al., 2020): the resonance peak at 1.2 ppm is attributed to the methyl group coupled to one proton; a doublet of quadruplet at 2.4 ppm is attributed to a methylene group adjacent to an asymmetric carbon atom bearing a single proton; and a multiplet at 5.2 ppm is characteristic of the methine group (Supplementary Material 4).

## Conclusion

In this work, a new microbial source of inulinase, *P. lanosocoeruleum*, was identified by screening a fungal library. Three potentially active inulinases, two related to the exoinulinase and one to the endoinulinase families, were identified in the *PlaI* enzymatic mixture.

The application of a statistical experimental design allowed to define the optimal conditions for inulin hydrolysis by *PlaI*, leading to envisage its exploitation as effective biocatalyst mixture for inulin processing.

The optimal conditions defined for the hydrolysis could be exported and incorporated into a process for industrial fructose syrup production, since in these conditions the formation of undesired color as well as the production of unwanted by products such as fructose dianhydrides are prevented (Mutanda et al., 2009). Additionally, information on the isoenzymes composition of the *PlaI* mixture represents the starting point for further characterization of the single isoenzymes, to be carried out through their purification from the culture broth or their recombinant expression in suitable hosts. The availability of each single isoenzyme will allow the formulation of inulinase mixtures with different composition in terms of endo- and exo- inulinase activities, addressing specific applications, such as the generation of fructose as well as of inulo-oligosaccharides (IOS) for applications as probiotics in food and pharmaceutical industries.

The integration of *PlaI*-catalyzed hydrolysis within a fermentation process finalized to the production of added-value bio-based products was tested, using PHA production as a case study. Two different process configurations, such as SHF and SSF, were explored, with the latter displaying the best performances in terms of biopolymer yields.

In conclusion, the results herein described let to foresee a profitable and versatile utilization of the *PlaI* mixture in inulin-based biorefineries.

## Data Availability Statement

The original contributions presented in the study are included in the article/Supplementary Material, further inquiries can be directed to the corresponding author.

## Author Contributions

IC: investigation, DOE experiments, PHA production, and formal analysis. NC: investigation, screening of fungal library, and inulinase characterization. GN: proteomic analysis. CP: conceptualization and writing of original draft. LB: supervision. GS: supervision and funding acquisition. All authors contributed to the article and approved the submitted version.

## Conflict of Interest

The authors declare that the research was conducted in the absence of any commercial or financial relationships that could be construed as a potential conflict of interest.

## Footnotes

1 https://www.jmp.com/it_it/home.html

2 https://genome.jgi.doe.gov/portal/PenlanEProfiling_FD/PenlanEProfiling_FD.info.html

3 www.matrixscience.com

4 http://www.matrixscience.com/help/interpretation_help.html

5 http://www.cazy.org/
