# Supplementary material for: Optimization of Inulin Hydrolysis by Penicillium lanosocoeruleum Inulinases and Efficient Conversion Into Polyhydroxyalkanoates
Source: Front Bioeng Biotechnol. 2021 Mar 1;9:616908. doi: 10.3389/fbioe.2021.616908 (PMC7959777; doi:10.3389/fbioe.2021.616908)
Supplement: Supplementary file 1 [file Table_1.docx]

**S1.** Significance values were estimated through Tukey-Kramer HSD test (p<0.05), comparing each mean to a control experiment that represents spontaneous inulin hydrolysis. Means that non share a letter are significantly different.

|  | Day | | | | | | | |
| --- | --- | --- | --- | --- | --- | --- | --- | --- |
|  | 2 | 3 | 4 | 5 | 6 | 7 | 9 | 10 |
| *Control* | 3.1 ^a^ | 3.1 ^a^ | 3.1 ^a^ | 3.1 ^a^ | 3.1 ^a^ | 3.1 ^a^ | 3.1 ^a^ | 3.1 ^a^ |
| *A. brasiliensis* | 3.4 ^b^ | 3.4 ^a^ | 5.2 ^d^ | 1.7 | 2.4 | 1.0 | 1.0 | 2.5 |
| *C. globosum* | 3.1 ^ab^ | 3.7 ^ab^ | 3.1 ^a^ | 2.9 | 3.5 ^ab^ | 2.4 | 2.4 | 3.7 ^a^ |
| *T. stipitatus* | 1.5 | 1.3 | 2.6 | 2.1 | 2.5 | 3.2 ^ab^ | 3.2 ^a^ | 3.2 ^a^ |
| *F. graminearum* | 3.6 ^d^ | 1.6 | 1.8 | 0.8 | 2.1 | 2.4 | 2.4 | 3.8 ^a^ |
| *T. lanuginosus* | 2.4 | 2.5 | 3.8 ^b^ | 3.0 | 3.9 ^b^ | 2.3 | 2.3 | 3.9 ^a^ |
| *P. canescens* | 4.0 ^e^ | 2.7 | 2.7 | 1.9 | 2.7 | 2.5 | 2.5 | 5.3 ^b^ |
| *P. expansum* | 3.5 ^bc^ | 4.6 ^bc^ | 4.6 ^c^ | 1.3 | 7.3 ^c^ | 3.2 ^ab^ | 3.2 ^a^ | 5.8 ^c^ |
| *H. haematococca* | 2.2 | 3.3 | 2.0 | 0.9 | 1.6 | 1.1 | 1.1 | 2.1 |
| *P. chrysogenum* | 4.2 ^f^ | 5.4 ^c^ | 3.8 ^b^ | 1.2 | 2.2 | 1.2 | 1.2 | 4.9 ^ab^ |
| *P. lanoscoeruleum* | 6.2 ^g^ | 12.0 ^d^ | 17.9 ^f^ | 17.4 ^b^ | 8.1 ^d^ | 8.5 ^c^ | 8.5 ^b^ | 4.8 ^ab^ |
| *P. brevicompactum* | 3.5 ^cd^ | 5.0 ^c^ | 6.6 ^e^ | 2.8 | 7.9 ^c^ | 2.6 | 2.6 | 2.2 |
| *P. raistrickii* | 2.2 | 1.3 | 1.6 | 1.6 | 1.8 | 1.9 | 1.9 | 1.5 |
