# Supplementary material for: Optimization of Inulin Hydrolysis by Penicillium lanosocoeruleum Inulinases and Efficient Conversion Into Polyhydroxyalkanoates
Source: Front Bioeng Biotechnol. 2021 Mar 1;9:616908. doi: 10.3389/fbioe.2021.616908 (PMC7959777; doi:10.3389/fbioe.2021.616908)
Supplement: Supplementary file 2 [file Table_2.DOCX]

**S2.** Identification of all *P. lanosocoeruleum* proteins in two active gel band lanes by LC-MS/MS analysis.

| **Lower gel band lane** | | | | | | | |
| --- | --- | --- | --- | --- | --- | --- | --- |
| **Protein code** | ***m/z*** | **Ion Score** | | | **Charge** | | **Peptide** |
| 323309 | 381.2071 | 17 | | | 2 | | R.SAMTVPR.K |
|  | 491.2646 | 47 | | | 2 | | K.VDGVHELGR.I |
|  | 493.8103 | 21 | | | 2 | | R.WVAVISLAK.L |
|  | 513.3074 | 45 | | | 2 | | K.AILVQEPAGK.W |
|  | 574.8382 | 38 | | | 3 | | K.VLIYTSPDLK.K |
|  | 639.3499 | 15 | | | 2 | | K.ALEINLTFSNR.E |
|  | 479.2473 | 26 | | | 3 | | R.YRPQFHFSPEK.N |
|  | 501.2348 | 28 | | | 3 | | R.DPGVFWHEETER.W |
|  | 826.4368 | 12 | | | 2 | | R.LFSTGGATSNVELEVK.E |
|  | 1051.546 | 23 | | | 3 | | K.ALEINLTFSNREPSPSSSSSEFGIVIAATK.G |
| 371227 | 404.2174 | 24 | | | 2 | | K.GMLSFPR.T |
|  | 461.7475 | 28 | | | 2 | | K.LWLDYGR.D |
|  | 504.2562 | 34 | | | 2 | | K.VFFHEGSGK.W |
|  | 587.3369 | 56 | | | 2 | | R.TLALQQTGSVR.S |
|  | 593.3001 | 15 | | | 2 | | K.VTFWTSTDTK.H |
|  | 609.8106 | 18 | | | 2 | | K.TFQPDPVDTAK.L |
|  | 685.8377 | 13 | | | 2 | | R.YNQSTSTLSVDR.T |
|  | 675.9683 | 17 | | | 3 | | R.DFDGAMSWENVPSSDGRR.I |
| 417764 | 468.2746 | 32 | | | 2 | | R.ASTFAIAVR.A |
|  | 492.2741 | 24 | | | 2 | | K.VIPTDPWR.S |
|  | 541.8222 | 45 | | | 2 | | K.VTLLQQPQR.S |
|  | 520.2441 | 19 | | | 3 | | K.IDLSFADHGPDESR.A |
| 376719 | 428.718 | 2 | | | 34 | | K.SSPGSFF.K |
|  | 451.2537 | 2 | | | 13 | | K.APSLDSAL.K |
|  | 498.7664 | 2 | | | 28 | | K.AFVIGSAMG.K |
|  | 555.2903 | 2 | | | 38 | | K.FTWGSIAEA.K |
|  | 426.8992 | 3 | | | 46 | | K.LDKEPGSYTI.R |
| 327740 | 433.7392 | 2 | | | 20 | | K.SITGPYT.K |
|  | 474.7482 | 2 | | | 28 | | R.GGSIDPAGF.K |
|  | 554.2916 | 2 | | | 49 | | R.VQVAVSDDF.K |
|  | 402.4562 | 4 | | | 31 | | R.GGSIDPAGFKDKDGS.R |
|  | 599.3307 | 3 | | | 5 | | K.VGADGVTPIGDAVQILD.R |
|  | 421.8962 | 3 | | | 32 | | K.RVQVAVSDDF.K |
| 383312 | 427.2345 | 2 | | | 31 | | K.EADIHL.R |
|  | 465.753 | 2 | | | 19 | | K.EIPISTD.R |
|  | 579.3351 | 2 | | | 21 | | K.GAVNIDLSQL.K |
|  | 582.282 | 2 | | | 16 | | K.EEQGVYPW.R |
|  | 602.8451 | 2 | | | 6 | | K.AIFSPVLSSGA.R |
|  | 424.2209 | 3 | | | 45 | | K.VWYGEHNLP.R |
|  | 512.2699 | 3 | | | 11 | | R.GAGHNFGIVTSATF.R |
| 387674 | 300.712 | 2 | | | 16 | | K.LVIQ.K |
|  | 336.6978 | 2 | | | 14 | | K.NGVNL.R |
|  | 381.7208 | 2 | | | 16 | | R.LAAAFN.R |
|  | 525.2803 | 2 | | | 36 | | R.LWFVQDN.K |
|  | 405.5417 | 3 | | | 33 | | R.ICHSVNIDT.R |
|  | 462.9207 | 3 | | | 16 | | K.DNAGWDKLVIQ.K |
| 383083 | 480.2723 | 2 | | | 19 | | K.KDWAAIQ.K |
|  | 527.3052 | 2 | | | 37 | | K.TPENILPAA.K |
|  | 538.79 | 2 | | | 27 | | K.NLLEEPSF.K |
|  | 630.8381 | 2 | | | 22 | | K.GVLVGTAESWN.K |
|  | 510.9559 | 3 | | | 2 | | K.VVLGMWVGKPTDT.K |
| 381496 | 539.2985 | 2 | | | 24 | | R.YGLAADQVLK |
|  | 491.5819 | 3 | | | 17 | | K.AKYDPNDIFYAR |
|  | 492.925 | 3 | | | 15 | | K.KVFYGTNYDTLR |
|  | 507.6094 | 3 | | | 11 | | K.VSGDVHNAVLPAWR |
|  | 521.6253 | 3 | | | 16 | | R.STGAGALAIWTHHLK |
| **Upper gel band lane** | | | | | | | |
| 371227 | 404.2158 | | 19 | 2 | | K.GMLSFPR.T | |
|  | 587.3391 | | 15 | 2 | | R.TLALQQTGSVR.S | |
|  | 609.8107 | | 14 | 2 | | K.TFQPDPVDTAK.L | |
|  | 658.334 | | 13 | 2 | | K.NWMNEPNGLIK.I | |
|  | 685.8384 | | 10 | 2 | | R.YNQSTSTLSVDR.T | |
|  | 727.8692 | | 12 | 2 | | R.LAYSVDDGVTWTK.F | |
|  | 935.4006 | | 13 | 2 | | R.DFDGAMSWENVPSSDGR.R | |
|  | 988.027 | | 18 | 2 | | R.ILAAIANSYGASPPTNTWK.G | |
|  | 764.0957 | | 28 | 3 | | R.SFVQWPVSELLTAGTALTTIR.N | |
|  | 847.0821 | | 37 | 3 | | K.FAGNPIISAAQEAPHDETDGLETR.D | |
| 384244 | 584.3059 | | 11 | 2 | | K.AVNFNVYAN.R | |
|  | 609.8239 | | 16 | 2 | | R.EWLLPNYQ.R | |
|  | 626.3339 | | 13 | 2 | | R.IAESILEDYA.K | |
|  | 659.3335 | | 19 | 2 | | K.LDQWAEETVA.R | |
|  | 782.4213 | | 15 | 2 | | R.ALMNTVSAFGAPIQ.K + Oxidation(M) | |
|  | 824.9556 | | 11 | 2 | | K.INFDIWNLIPFT.R | |
|  | 574.6376 | | 21 | 3 | | R.GVSMIPNNLHENQI.R | |
|  | 892.9588 | | 33 | 2 | | K.FFMNELDLLGQAAAT.K + Oxidation(M) | |
|  | 935.5109 | | 20 | 2 | | K.TYDYVIAGGGLTGLTVAA.K | |
|  | 729.7245 | | 31 | 3 | | K.GIELLDT.KLDQWAEETVA.R | |
|  | 760.3507 | | 15 | 3 | | K.VFGMEGWNWDNVFQYMQ.K | |
|  | 1145.576 | | 20 | 2 | | R.GSVHILSSDPYLWQYANDP.K | |
|  | 777.4545 | | 27 | 3 | | R.EVLLAAGSSISPLILEYSGIGI.K | |
|  | 787.0504 | | 27 | 3 | | R.NWLLDEDVAFAELFFDTEG.K | |
|  | 871.8002 | | 51 | 3 | | K.AGVEQLLELPVGLNMQDQTTTTV.R | |
|  | 677.1094 | | 11 | 4 | | R.VIDGSIPPTQVSSHVMTVFYGMAL.R | |
|  | 779.9056 | | 21 | 4 | | K.SGLGLGGSTLINGDSWT.RPD.KVQIDSWE.K | |
|  | 1052.57 | | 44 | 3 | | K.SVLDKAGVEQLLELPVGLNMQDQTTTTV.R | |
|  | 830.1417 | | 15 | 4 | | R.S.RPPTDAQIEAGHFYDPACHGTDGTVHAGP.R | |
| 315441 | 404.2388 | | 13 | 2 | | R.FVVDVT.K | |
|  | 415.7339 | | 19 | 2 | | K.NLNGVW.K | |
|  | 618.3332 | | 13 | 2 | | R.QLDLMVNQF.K | |
|  | 670.8417 | | 11 | 2 | | K.EAMVYDLQML.K + Oxidation(M) | |
|  | 743.853 | | 16 | 2 | | K.LGDDQVESYTGF.R | |
|  | 641.6876 | | 20 | 3 | | K.VGTNPWPEYP.RPLLQ.R | |
|  | 667.6922 | | 14 | 3 | | R.SPGYAL.KEPPLTTPWTD.K | |
|  | 782.0635 | | 26 | 3 | | R.DANGNSVTILPNEQQQIEFA.R | |
|  | 834.0953 | | 29 | 3 | | R..RDANGNSVTILPNEQQQIEFA.R | |
|  | 978.49 | | 26 | 3 | | K.FDQSNELLVFVHDPTDDGDYVIPIG.K | |
|  | 1008.137 | | 12 | 3 | | R.LYACSGGVWTQTTDVEGEVNGLMTYD.R | |
|  | 1158.919 | | 41 | 3 | | K.SFPSIVTWVIYNEGWGQITAYNPEFALTD.R | |
|  | 880.9503 | | 40 | 4 | | K.IETDLFYQACDELGLLVIQDMPSL.RPLQS.R | |
|  | 1176.264 | | 30 | 4 | | R.LVDSTSGWVDHGAGDFSDNHHYANPQCGSPFYSTPSSPYDSS.R | |
| 400960 | 742.9103 | | 19 | 2 | | R.NIDVDPITINGVS.K | |
|  | 975.9612 | | 20 | 2 | | R.AQDMNNIASWTDEINT.K | |
|  | 722.0639 | | 17 | 3 | | K.FTSNGIIPPAITGLHNGDAL.R | |
|  | 805.4335 | | 12 | 3 | | R.LAEWPIITLTHQEMSASFLA.R | |
|  | 852.7726 | | 17 | 3 | | K.IAAFAPTTGFTTESVAGVINNFDG.R | |
|  | 903.7664 | | 15 | 3 | | K.ENAMWPLFTTAAANNFDGMQINP.R | |
|  | 1458.703 | | 35 | 2 | | R.VNLNTQIDDMFLETEIYSPAGENF.R | |
|  | 1024.839 | | 21 | 3 | | R.RVNLNTQIDDMFLETEIYSPAGENF.R | |
|  | 1127.202 | | 41 | 3 | | K.FGTSAIGGCCDNGVEQLVSFTDISDFPTSGL.K | |
|  | 1217.258 | | 60 | 3 | | R.IYYNCDTPACTVAEWIATSAGAGTFQDLLAIE.K | |
| 385992 | 371.7485 | | 14 | 2 | | K.AGALLLG.K | |
|  | 355.1764 | | 15 | 3 | | R.DSYVVHGM.R | |
|  | 631.3589 | | 16 | 2 | | R.GYLERPLPVAF | |
|  | 484.9088 | | 11 | 3 | | R.DYLSEVENTNI.R | |
|  | 509.9729 | | 18 | 3 | | R.NSVVGIKPTVGLTS.R | |
|  | 601.3202 | | 14 | 3 | | R.METTAGSWALLGNVVP.R | |
|  | 691.6985 | | 25 | 3 | | K.DRMETTAGSWALLGNVVP.R | |
|  | 856.4602 | | 20 | 3 | | R.VHQTQPYLNAILQVNPDAFSIA.K | |
|  | 1020.547 | | 24 | 3 | | K.GAVFGIPWESFWALGDADQIAQLLDLV.K | |
|  | 1383.357 | | 46 | 3 | | R.SVEDLVQYNIDNYGSEGGLPGIHPAFGSGQDGLLASLET.K | |
| 323309 | 381.2074 | | 11 | 2 | | R.SAMTVP.R | |
|  | 327.8467 | | 14 | 3 | | K.VDGVHELG.R | |
|  | 604.8002 | | 14 | 2 | | K.TTGDFVDASPA.K | |
|  | 426.2597 | | 14 | 3 | | K.VLIYTSPDL.K.K | |
|  | 768.8942 | | 12 | 2 | | R.ANQQAQSIAYSLD.K | |
|  | 822.9132 | | 15 | 2 | | R.VGYNFGTQEVFID.R | |
|  | 826.4343 | | 10 | 2 | | R.LFSTGGATSNVELEV.K | |
|  | 1260.625 | | 12 | 2 | | R.TVIAWMNNWQYGATIPTDPW.R | |
|  | 1001.834 | | 32 | 3 | | R.SGTIEIIDNATGGWGHLNVDEISFSNT.R | |
|  | 1101.858 | | 33 | 3 | | R.ANNQVANWLDWGPDFYAALGWNGLPQDD.R | |
|  | 1118.878 | | 12 | 3 | | K.GMTWTTYDAANPVIPEPPAPYQDQFLEF.R | |
|  | 1154.906 | | 19 | 3 | | K.WGLASEFGPVNAVGGVWECPSIFPLSLDGGES.K | |
| 373946 | 357.7337 | | 12 | 2 | | K.AQAALL.K | |
|  | 672.3169 | | 11 | 2 | | K.ANVASVMCSYN.K | |
|  | 480.9267 | | 14 | 3 | | K.ATVDVTADHASVV.R | |
|  | 524.3142 | | 15 | 3 | | K.GLGVHVQLGPAAGPLG.K | |
|  | 607.3166 | | 18 | 3 | | R.ATHELYLWPFMNAV.K | |
|  | 698.0213 | | 17 | 3 | | R.YANPVTAFPAGINAGATWD.R | |
|  | 757.0534 | | 25 | 3 | | K.SLAIIGQDAIVNPDGANACVD.R | |
|  | 842.5154 | | 31 | 3 | | K.NVIVVVHSVGPIILETILAQPSV.K | |
|  | 967.8222 | | 7 | 3 | | R.ILAAWYLLGQDQGYPEVTFSSWDGG.K | |
|  | 994.7978 | | 29 | 3 | | K.QSSDYGAGWDSALVDNFVEDLFIDY.R | |
|  | 1005.164 | | 8 | 3 | | R.GCNTGTLAMGWGSGTSEFPYLVAPLDAI.K | |
| 383654 | 460.7505 | | 14 | 2 | | K.YGVPLDT.R | |
|  | 682.8629 | | 11 | 2 | | R.SPYLSTWLNVG.K | |
|  | 779.3626 | | 11 | 2 | | K.DYTESSNLASSLD.R | |
|  | 551.6083 | | 18 | 3 | | K.SGDSGFLNDHYSLL.K | |
|  | 886.9118 | | 10 | 2 | | K.GDWECFTAAVSSVST.R | |
|  | 670.9875 | | 17 | 3 | | R.NAIAEWEYGSTDGVAYH.K | |
|  | 1040.523 | | 21 | 2 | | R.AMFISDLATWINETPTN.R | |
|  | 785.3874 | | 15 | 3 | | R.QAFGATQLCGT.KD.KMYMFL.K | |
|  | 633.8404 | | 23 | 4 | | R.RVEHDSIATAGQEYLTVTSLSV.R | |
|  | 873.4398 | | 18 | 3 | | R.AISSSWPVFGFSYNLGSVDSSPVS.K | |
|  | 1133.925 | | 21 | 3 | | K.EISSNGNMNTVDVIFPAYPIFLYTNPELL.K | |
| 401322 | 527.3255 | | 10 | 2 | | K.GGLIIEQPV.K | |
|  | 404.2158 | | 13 | 3 | | K.IEDAPLYPH.R | |
|  | 672.3646 | | 16 | 2 | | R.AAALGELVWSGN.R | |
|  | 562.0702 | | 18 | 4 | | K.GGLIIEQPVKIEDAPLYPH.R | |
|  | 918.5099 | | 33 | 3 | | R.INAQTVWGVLHAFTTLQQIIISDG.K | |
|  | 975.5015 | | 22 | 3 | | K.HIVGAEAPLWSEQVDDVTVSSVFWP.R | |
|  | 782.8748 | | 17 | 4 | | K.LNVLHWHLDDSQSWPVQMSSYPEMT.K | |
| 378182 | 551.2947 | | 19 | 2 | | R.AVEQSLDAI.R | |
|  | 575.7995 | | 13 | 2 | | K.AFDSEFPLP.K | |
|  | 688.8705 | | 16 | 2 | | K.FGQAIAAGSDAQL.K | |
|  | 702.3894 | | 18 | 2 | | K.NVGFPVLSVTEN.K | |
|  | 819.9089 | | 18 | 2 | | R.AGMIADSGALAASGYQ.R | |
|  | 546.9513 | | 17 | 3 | | R.ERDFPIPDLDFF.K | |
|  | 758.4009 | | 13 | 3 | | R.TGDVRPEEDQTLFPVMLGL.R | |
|  | 902.4756 | | 14 | 3 | | R.FLRTGDVRPEEDQTLFPVMLGL.R | |
| 327740 | 898.4892 | | 19 | 2 | | K.VGADGVTPIGDAVQILD.R | |
|  | 947.9703 | | 16 | 2 | | K.GAWLGLNTNFPDPSFM.K | |
|  | 704.3441 | | 19 | 4 | | R.HHCVGTAIADHPAGPYVPSNTPLSC.R | |
|  | 1171.246 | | 25 | 3 | | K.SWTLLDVEALPTLSTWETENDHWAPDVVM.R | |
