# Supplementary material for: Optimization of Inulin Hydrolysis by Penicillium lanosocoeruleum Inulinases and Efficient Conversion Into Polyhydroxyalkanoates
Source: Front Bioeng Biotechnol. 2021 Mar 1;9:616908. doi: 10.3389/fbioe.2021.616908 (PMC7959777; doi:10.3389/fbioe.2021.616908)
Supplement: Supplementary file 3 [file Table_3.docx]

**S3.** Analysis of variance of fitted model

| **Source** | **Sum of squares** | **Degrees of freedom** | **Mean square** | **F Value** | **p Value Prof>F** |
| --- | --- | --- | --- | --- | --- |
| **Model** | 2017.1 | 14 | 144.1 | 73.42 | <.0001* |
| **pH (A)** | 420.9 | 1 | 420.9 | 214.5 | <.0001* |
| **°C (B)** | 143.7 | 1 | 143.7 | 73.2 | <.0001* |
| **Enzyme concentration (C)** | 214.7 | 1 | 214.7 | 109.4 | <.0001* |
| **Substrate Concentration (D)** | 410.1 | 1 | 410.1 | 208.98 | <.0001* |
| **AB** | 40.2 | 1 | 40.2 | 20.47 | 0.0007* |
| **AC** | 1.96 | 1 | 1.96 | 1.00 | 0.3374 |
| **BC** | 1.7 | 1 | 1.7 | 0.87 | 0.3704 |
| **AD** | 7.89 | 1 | 7.89 | 4.02 | 0.0681 |
| **BD** | 21.1 | 1 | 21.1 | 10.75 | 0.0066* |
| **CD** | 1.15 | 1 | 1.15 | 0.59 | 0.4583 |
| **A^2^** | 177.5 | 1 | 177.5 | 90.46 | <.0001* |
| **B^2^** | 443.8 | 1 | 443.8 | 226.16 | <.0001* |
| **C^2^** | 1.06 | 1 | 1.06 | 0.54 | 0.4766 |
| **D^2^** | 19.6 | 1 | 19.6 | 9.98 | 0.0082* |
| **R-squared** |  |  |  |  | 0.98 |
| **Adj squared** |  |  |  |  | 0.97 |
| **R-squared (pred)** |  |  |  |  | 0.93 |
