# Supplementary figures and images for: Optimization of Inulin Hydrolysis by Penicillium lanosocoeruleum Inulinases and Efficient Conversion Into Polyhydroxyalkanoates
Source: Front Bioeng Biotechnol. 2021 Mar 1;9:616908. doi: 10.3389/fbioe.2021.616908 (PMC7959777; doi:10.3389/fbioe.2021.616908)

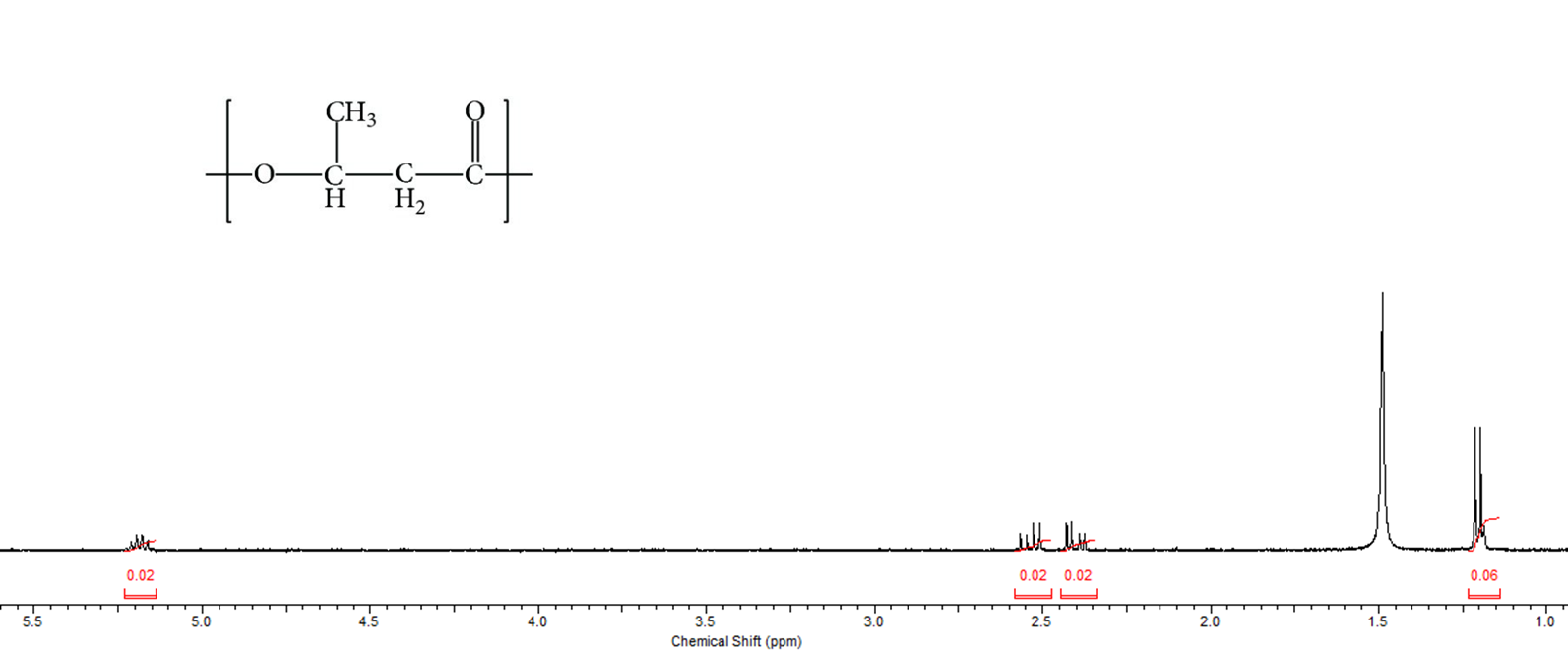
 **S4.** 1H NMR spectra of PHA extract

Supplement: Supplementary file 4 [file Table_4.DOCX]
